# Impact of Periodic Follow-Up Testing Among Urban American Indian Women With Impaired Fasting Glucose

**Published:** 2008-06-15

**Authors:** Peg Allen, Janice L Thompson, Carla J Herman, Ayn N Whyte, Venita K Wolfe, Clifford Qualls, Deborah L Helitzer

**Affiliations:** Division of Geriatrics, Department of Internal Medicine, School of Medicine, University of New Mexico Health Sciences Center; Office of Native American Diabetes Programs, Department of Internal Medicine, School of Medicine, University of New Mexico Health Sciences Center, Albuquerque, New Mexico. Dr. Thompson is now with the Centre for Sport, Exercise and Health, Department of Exercise and Health Sciences, University of Bristol, England; Office of Native American Diabetes Programs, Department of Internal Medicine, School of Medicine, University of New Mexico Health Sciences Center, Albuquerque, New Mexico. Dr. Herman is now with the Division of Geriatrics; Office of Native American Diabetes Programs, Department of Internal Medicine, School of Medicine, University of New Mexico Health Sciences Center, Albuquerque, New Mexico. Ms. Whyte is now with the New Mexico Tumor Registry, School of Medicine, University of New Mexico Health Sciences Center, Albuquerque, New Mexico; Office of Native American Diabetes Programs, Department of Internal Medicine, School of Medicine, University of New Mexico Health Sciences Center, Albuquerque, New Mexico. Ms. Wolfe is now with the Division of Geriatrics; General Clinical Research Center, Department of Internal Medicine, School of Medicine, University of New Mexico Health Sciences Center, Albuquerque, New Mexico. Dr. Qualls is now with the Clinical and Translational Science Center; Health Evaluation and Research Office, Department of Family and Community Medicine, School of Medicine, University of New Mexico Health Sciences Center, Albuquerque, New Mexico. Dr. Helitzer is now with the Department of Family and Community Medicine

## Abstract

**Introduction:**

Impaired fasting glucose (IFG) often progresses to type 2 diabetes. Given the severity and prevalence of this disease, primary prevention is important. Intensive lifestyle counseling interventions have delayed or prevented the onset of type 2 diabetes, but it is not known whether less intensive, more easily replicable efforts can also be effective.

**Methods:**

In a lifestyle intervention study designed to reduce risks for type 2 diabetes, 200 American Indian women without diabetes, aged 18 to 40 years, were recruited from an urban community without regard to weight or IFG and block-randomized into intervention and control groups on the basis of fasting blood glucose (FBG). Dietary and physical activity behaviors were reported, and clinical metabolic, fitness, and body composition measures were taken at baseline and at periodic follow-up through 18 months. American Indian facilitators used a group-discussion format during the first 6 months to deliver a culturally influenced educational intervention on healthy eating, physical activity, social support, and goal setting. We analyzed a subset of young American Indian women with IFG at baseline (n = 42), selected from both the intervention and control groups.

**Results:**

Among the women with IFG, mean FBG significantly decreased from baseline to follow-up (*P* < .001) and converted to normal (<5.6 mmol/L or <100 mg/dL) in 62.0% of the 30 women who completed the 18-month follow-up, irrespective of participation in the group educational sessions. Other improved metabolic values included significant decreases in mean fasting blood total cholesterol and low-density lipoprotein cholesterol levels. The women reported significant overall mean decreases in intake of total energy, saturated fat, total fat, total sugar, sweetened beverages, proportion of sweet foods in the diet, and hours of TV watching.

**Conclusion:**

Volunteers with IFG in this study benefited from learning their FBG values and reporting their dietary patterns; they made dietary changes and improved their FBG and lipid profiles. If confirmed in larger samples, these results support periodic dietary and body composition assessment, as well as glucose monitoring among women with IFG.

## Introduction

Impaired fasting glucose (IFG) left unattended often progresses to type 2 diabetes (1-4). With the alarming rise in type 2 diabetes in the United States and internationally, and with younger ages at onset (5,6), it is increasingly urgent that we identify and assist people with IFG in lowering blood glucose levels before insulin resistance leads to beta cell failure.

IFG is defined as fasting blood glucose (FBG) of 5.6–6.9 mmol/L (100–125 mg/dL) (1). On the basis of this definition, the 1999–2002 National Health and Nutrition Examination Survey (NHANES) indicated a 9.3% prevalence of diabetes (diagnosed and undiagnosed) and 26.0% prevalence of IFG among U.S. adults aged 20 years and older (7). In this same survey, 8.2% of women aged 20 to 39 and 7.3% of girls aged 12 to 19 had IFG (6).

Rates of type 2 diabetes are high among American Indians and Alaska Natives compared with other U.S. groups (8). NHANES did not report national estimates of IFG specifically for American Indians (6,7), but a study of 1056 Alaska Eskimos aged 18 and older found that 15.6% of all adults and 13.9% of women had IFG (9). The Inter-Tribal Heart Project found 16.8% of 1376 American Indian adults (aged 25 and older) without diabetes had FBG values of 6.1–6.9 mmol/L (110–125 mg/dL) (10).

Risk factors for IFG, impaired glucose tolerance (IGT), and type 2 diabetes include obesity or overweight, sedentary lifestyle, family history, and consumption of foods high in saturated fat and added sugars (11-13). In 2002–2004, an estimated 33.0% of American Indian and Alaska Native women were obese (body mass index [BMI] ≥30.0 kg/m^2^) and 23.3% were overweight (BMI 25.0–29.9 kg/m^2^), compared with 23.2% and 27.3% of U.S. women overall, respectively (5). An estimated 45.5% of American Indian and Alaska Native women report no leisure-time physical activity, compared with 39.8% of U.S. women overall (5). Intensive individual lifestyle counseling interventions among adults older than 50 years with IGT have resulted in dietary changes, increased physical activity, and weight loss and have delayed or prevented the onset of type 2 diabetes (4,13). What is not known is whether less intensive, more easily replicable lifestyle interventions can reduce risk for type 2 diabetes among young adults (14).

The purpose of this study was to conduct an analysis of results among a small subset of women with IFG at baseline who participated in a lifestyle intervention study with an 18-month follow-up. The larger study tested efficacy of a low-intensity lifestyle intervention to reduce risks for type 2 diabetes among 200 urban American Indian women aged 18 to 40 recruited from the general community without regard to risk factors for type 2 diabetes. Results of the randomized controlled trial are reported elsewhere (15).

## Methods

### Participants

American Indian women were recruited from June 2002 through June 2004 from a southwestern U.S. city to participate in a randomized controlled trial to test the effectiveness of a healthy lifestyle intervention in reducing risks for type 2 diabetes. Participants were recruited via word of mouth; flyers posted at outpatient clinics, colleges, and major employers; and local media. Eligibility criteria included self-identification as American Indian, female, aged 18–40 at baseline, without diabetes, planning to stay in the local area for 2 years, not pregnant, and not planning a pregnancy in the next 2 years. Human subjects approval was obtained from the University of New Mexico Health Sciences Center Human Research Review Committee and the local Indian Health Service clinic. Potential volunteers were screened over the phone for eligibility; eligible women were invited to an in-person meeting for further explanation of the study and to obtain written informed consent. To be included in the study, the 200 women had to be without diabetes at baseline as measured by FBG <7.0 mmol/L (<126 mg/dL) (1).

### Randomization

The 200 eligible volunteers were block-randomized by FBG level into 2 groups of 100 women each (intervention and control) to ensure equivalent representation among the 2 groups. The intervention was implemented between the baseline and 6-month clinic measures, with follow-up clinic measurements at 12 and 18 months. Women from the control group completed the 4 clinic measurements at baseline and at 6, 12, and 18 months and were then offered and provided the same intervention.

### Intervention

A multidisciplinary and multiethnic team of American Indian and non-Indian health professionals and community members drafted a 5-session curriculum and pilot-tested it with members of the target community (16). The healthy lifestyles intervention consisted of discussion-format group sessions that took place once a month and were facilitated by female American Indian staff. The intervention included written and oral didactic material with culturally appropriate graphics, and participation in small-group discussions. The first session covered diabetes-related definitions and the importance of physical activity. Subsequent sessions promoted eating more vegetables and fruits and less saturated fat and added sugar, setting goals, getting social support for behavior change, and maintaining behavior change.

### Measurements

Trained dietetics and nursing staff conducted the baseline and follow-up clinical measurements at the University of New Mexico Health Sciences Center outpatient General Clinical Research Center (funded by the National Institutes of Health), from June 2002 through February 2006. After an overnight fast, volunteers had their blood drawn via venipuncture and analyzed for glucose, insulin, and lipids. Insulin sensitivity was estimated by using the quantitative insulin sensitivity check index: 1/(log insulin + log glucose) (17). Blood chemistry analysis methods are described elsewhere (18). Participants' height, weight, and hip and waist circumferences were measured by using standard methods. Body composition was measured with Quantum bioelectrical impedance software (RJL Systems, Clinton Township, Michigan), using a prediction equation validated with American Indian women (19). After each clinic visit, each participant was informed in writing of her results and the expected and at-risk ranges for FBG, lipids, body composition, and blood pressure. Participants were encouraged to share these results with their primary care providers.

The women also reported previous 6-month food and beverage intake on the Block Food Frequency Questionnaire (20) plus a supplemental form on Southwestern foods previously validated in New Mexico (21). A 24-hour dietary recall was also administered at each clinic visit. History of gestational diabetes and family history of type 2 diabetes was self-reported as yes/no at the first clinic visit.

Self-reported physical activity over the previous year at baseline, and over the previous 6 months during the study period, was assessed by using the Modifiable Activity Questionnaire (22); this tool has been tested for reliability and validity among Pima Indian adults. Predicted peak oxygen consumption was obtained during a submaximal bicycle ergometer test with the YMCA protocol (23).

### Statistical analyses

We conducted analyses for the subset of 42 women with IFG at baseline (FBG ≥5.6 mmol/L or ≥100 mg/dL), 19 of whom were in the intervention group and 23 in the control group. Mean changes at follow-up visits were obtained by subtracting each woman's baseline value from the value at each subsequent time point; differences were analyzed by using one-way repeated measures analyses of variance (RM ANOVA). Post hoc comparisons of mean changes at each time point from baseline were obtained by using paired *t* tests. Initially, group by visit (2x4) RM ANOVA were conducted to detect possible differences in means due to intervention. However, the sample was too small to detect between-group differences (data not shown). Therefore, we conducted pooled analyses that disregarded intervention group status.

## Results

### Baseline

Of the 200 eligible women, 42 (21.0%) had IFG at baseline. Baseline results comparing the 42 women with IFG and the 158 with normal FBG are reported elsewhere (18). At baseline, women with IFG were significantly older, with a higher mean BMI, waist circumference, percentage of body fat, fasting insulin, triglycerides, and diastolic blood pressure, and lower mean high-density lipoprotein (HDL) cholesterol than women with normal FBG (data not shown). Table 1 shows the baseline characteristics of the 42 women with IFG.

### Follow-up

Retention at the 18-month clinic visit was 30 (71.4%) of the original 42 women with IFG. Reasons for dropout included moving out of the area, pregnancy, or perception of being too busy to continue.

### Fasting blood glucose

Table 2 and Table 3 show mean baseline values and mean changes in metabolic, body composition, dietary, and physical activity measurements at follow-up. Women with IFG had significant mean reductions in FBG over time (*P* < .001) (Table 2). Their FBG at 6 months was a mean of 3.7% lower than baseline FBG, and at 12 and 18 months, respectively, was 3.2% and 6.6% lower. Of the women who completed follow-up, 62% converted to normal FBG by 18 months. Insulin sensitivity remained stable throughout the study despite reductions in FBG (data not shown).

### Lipids

Mean lipid profiles were within normal ranges at baseline. Nevertheless, total cholesterol and low-density lipoprotein (LDL) cholesterol significantly decreased among all women with IFG over time. Changes in HDL over time were not significant. By 12 months there was a 6.3% increase in HDL among the women with IFG, but by 18 months the increase from baseline was 4.5% of the baseline value (*P* = .07).

### Weight and waist circumference

Although overall mean changes in BMI, weight, and waist circumference were small (Table 2), more than half of the women weighed less at each follow-up visit than they did at baseline (51.5% at 6 months, 51.5% at 12 months, and 53.3% at 18 months) (data not shown). Among women who lost weight, the average percentage of baseline weight lost was 3.5% (SD, 3.3%) at 6 months, 6.0% (SD, 5.8%) at 12 months and 6.2% (SD, 4.9%) at 18 months. Women who lost weight at 18 months reduced their FBG by an average of 12.0% of baseline value at 18 months, and women without weight loss at 18 months had a 1.1% reduction in FBG (*P* = .002). At 18 months, 54.8% of the women lost waist circumference compared to baseline, with a mean reduction among these women of 5.4% (SD, 4.2%) of baseline waist circumference (data not shown).

### Other changes at follow-up

Over time women with IFG significantly decreased mean total energy intake, total fat intake, saturated fat intake, total sugar intake, proportion of sweet foods in the diet, and intake of sweetened beverages (Table 3). Both mean total fat and saturated fat intakes decreased by approximately one-fourth at 18 months. Fitness levels from the bicycle test remained stable throughout the study, as did blood pressure. Mean number of hours spent watching television significantly decreased over time. The average reported amount of time spent in leisure physical activity was 4.4 hours (SD, 3.8 hours) per week at baseline and did not change significantly over time. However, 43.3% of the women who completed follow-up reported physical activity at 18 months that totaled an increase of more than 2.0 hours/week from baseline (data not shown).

## Discussion

### Implications for screening and periodic follow-up

Results from this post hoc subset analysis show that once these volunteers learned they had IFG, many were successful in making dietary changes that resulted in weight loss, lower FBG, and better lipid profiles, regardless of whether they participated in the low-intensity intervention or just the follow-up clinical measures. Of the women who completed follow-up, 62% converted to normal FBG by 18 months. The FBG screening and periodic follow-up visits appear to have been an unintended intervention. Seeing a research dietitian at each clinic visit may have raised the women's awareness of their dietary patterns and body composition. Women received their clinical results after each visit and thus could track their progress over the follow-up period. This information was shared with participants because of the importance of full disclosure to this high-risk group. These results suggest that periodic follow-up that includes more than just blood glucose screening may improve metabolic control more than does follow-up with blood glucose screening alone. Although these findings need to be confirmed in larger samples, they imply that interventions should include regular dietary assessment and measurement of body composition, in addition to glucose monitoring.

The American Diabetes Association (ADA) recommends FBG screening or an oral glucose tolerance test every 3 years to detect IFG, IGT, or undiagnosed diabetes in all adults aged 45 years and older, especially if overweight, and more frequent screening tests if additional risk factors are present (1). Among adults younger than 45, ADA recommends screening those who are overweight (BMI ≥25.0 kg/m^2^) and have additional risk factors for diabetes, including physical inactivity, family history of diabetes, belonging to an ethnic group with a high prevalence of diabetes, history of gestational diabetes, high blood pressure, low HDL cholesterol, or high triglycerides (1).

The extent to which ADA screening and follow-up are implemented among American Indians is a concern, since this population has a high rate of type 2 diabetes and its complications. Participants' comments and questions indicated that many of these urban-dwelling American Indian women had not previously been screened for type 2 diabetes. Many tribes around the country have recently initiated or expanded diabetes prevention services in tribal communities. A recent telephone interview study in Montana found 72% of 428 randomly selected American Indian adults aged 18 to 44 years living on or near a reservation recalled having a blood glucose screening for diabetes within the past 3 years (24), but data on the extent of screening and monitoring for urban American Indian adults are lacking.

Urban American Indians are a growing population that is under-served for preventive health care; in the 2000 U.S. census, 61% (1.5 million) of Americans who reported American Indian or Alaska Native ethnicity alone lived in urban areas (25). Of these, only 34% resided in counties with a federally funded urban outpatient clinic serving American Indians, and these clinics nearly lost their federal funding in a recent budget cycle (25). ADA recommends that FBG or IGT screening be conducted only as part of a health care office visit (1). Fully implementing ADA screening recommendations among urban American Indian and other high-risk adults presents many challenges, particularly 1) identifying a health care system that has the infrastructure and ongoing funding to reach these residents and 2) providing follow-up monitoring (and ideally, support for lifestyle changes) after diagnosing adults with IFG or IGT, which can carry emotionally laden or negative labels. However, added impetus to resolve these challenges may come from the growing body of evidence that IFG and IGT (before progression to type 2 diabetes) may increase the risk for hypertension, abnormal blood lipid profiles, and cardiovascular diseases (26,27).

During study design, the research team decided to disclose and explain results of each clinic visit in writing to participants because of the seriousness of type 2 diabetes and potential to prevent or delay the disease, despite potential contamination of the research findings. This disclosure likely contributed to the lack of significant difference between the control and intervention groups. If findings from this study are confirmed in larger samples, periodic follow-up that promotes specific client awareness of clinical results, along with detailed dietary assessments, may be an effective low-intensity intervention with high-risk women. Because of the ethical principle of not withholding effective interventions, researchers in future studies might then be ethically bound to disclose future study participants' blood glucose and other markers.

### Low-intensity vs high-intensity interventions

The major clinical trials that used intense lifestyle interventions with frequent participant contact produced changes in physical activity that this study did not and found larger mean physiologic changes and similar mean self-reported dietary changes (4,11,13,28,29). The high baseline average reported amount of time spent in leisure physical activity of 4.4 hours (SD, 3.8 hours) per week may partially explain the lack of significant increase in physical activity here. More intense interventions may be needed to stimulate increased participation in moderate- and vigorous-intensity activity (30).

At the end of the 3-year Diabetes Prevention Program (DPP) lifestyle intervention study, participants had attended an average of 50.3 sessions (SD, 21.8 sessions), and 27% of adults younger than 45 met the 7% weight loss goal, compared with 63% of those 65 and older (29). Weight loss was not a direct aim of the intervention in the present study, so no target weight loss goal was identified. However, 20% of women who completed follow-up lost at least 7.0% of baseline weight at 18 months (data not shown). Lipid profile improvements were smaller in this study than those reported in a review of intense lifestyle interventions (13), but mean baseline lipid profiles were near normal in the present study.

Lower fat intake, particularly saturated fat intake, may protect against development of type 2 diabetes (11). Baseline dietary fat and saturated fat intakes were similar in the present study to those of the American Indian women in the DPP and other studies (31,32). At 1 year, the 45 American Indian women in the DPP lifestyle intervention reported a reduction in percentage of energy from total fat of 4.3% (SD, 6.8%), with a concomitant reduction in percentage of energy from saturated fat of 2.0% (SD, 3.4%). We found greater reductions in fat and saturated fat intake in the present study at 12 months, but weight loss was of lower magnitude.

Intake of sweetened beverages is linked to weight gain and to development of type 2 diabetes (11,12,33). The general U.S. population has increased its intake of these beverages over the past 20 years (34). Although we did not find national results for American Indian women specifically, other studies indicate this population also has a high intake of sweetened beverages (35,36). Among a convenience sample of 203 urban American Indian women in Minnesota (mean age 33.8 years), 66.7% reported drinking at least 1 soda with sugar per day, and half reported drinking Kool-Aid daily (37). At 1-year follow-up, the 45 American Indian women in the lifestyle intervention arm of the DPP study reduced their intake of sweets by a mean of 7.3 total servings/week (SD, 17.4 servings/week). In the present study, at 1 year total percentage of sweets in the diet decreased 3.7% (SD, 10.5%) and intake of sweetened beverages decreased 7.2 ounces/day (SD, 20.1 ounces/day).

A review of 7 low-intensity community-based lifestyle interventions designed to reduce risk for type 2 diabetes among adults (14) found increased knowledge about physical activity and nutrition, and increased self-reported physical activity levels. Two of the interventions also showed decreased waist circumference or BMI. Most did not include clinical measures other than weight. Interventions varied, lasting from 6 to 16 months, but most involved walking groups and/or group educational sessions and cooking demonstrations. Providing structured physical activities likely could have benefited women in the present study.

### Limitations

This subset analysis from a larger study has several limitations. Statistical power was not great enough to detect differences in mean changes between women with IFG in the intervention and control groups because of the small sample size and the wide variability in the women's dietary and metabolic values. Had this study been designed after DPP results were known, low-risk women might well have been excluded, creating a larger sample size of women with IFG. A tendency to self-report intervention-related and socially desirable dietary changes has been well-documented (38), but the reported dietary changes here were supported by actual weight loss. Previous authors have suggested American Indian dietary patterns may appear similar to each other and to the overall U.S. population because of incomplete recording of traditional foods or seasonal foods consumed at ceremonies, and other factors (39). To minimize these issues we used a supplemental set of questions to capture traditional foods and collected data at different times of the year. The study volunteers may not be representative of other similarly aged urban American Indian women or other populations. Participants may have volunteered because of family history of type 2 diabetes or other concerns about their health status as well as readiness to make lifestyle changes. A further limitation is that a single FBG measurement is not as accurate an estimate of impairment in the body's processing of glucose as are repeated FBG measurements or the 2-hour oral glucose tolerance test. However, the current study design and resources did not allow for repeat FBG testing at each measurement period.

### Conclusion

The DPP and other lifestyle interventions that involve frequent contact with high-risk adults are effective, but such intensive repeated one-on-one or even group counseling is expensive and challenging to replicate in communities and in typical health care settings (40). It is encouraging that many of the women with IFG in the present study made dietary changes, lost weight, improved lipid profiles, and converted to normal FBG after infrequent FBG monitoring and other clinical and dietary assessments. We hope the experiences of this group of women will inspire other researchers to conduct tests with larger samples to determine whether periodic, low-intensity, fully disclosed monitoring of dietary patterns, body composition, FBG, and other markers can help high-risk adults delay or prevent the onset of type 2 diabetes.

## Figures and Tables

**Table 1:** Baseline Participant Characteristics Among 42 Urban American Indian Women With Impaired Fasting Glucose From a Southwestern U.S. City, 2002–2004

Characteristic	Baseline Value
**Mean age, y (SD)**	31.6 (6.0)
**Body mass index (BMI) category, n (%)**	
Obese (BMI ≥30.0 kg/m^2^)	30 (71.4)
Overweight (BMI 25.0-29.9 kg/m^2^)	10 (23.8)
BMI <25.0 kg/m^2^	2 (4.8)
**Family history of type 2 diabetes, n (%)**	33 (80.5)
**History of gestational diabetes, n (%)**	3 (7.1)
**Education, n (%)**	
College graduate	6 (16.2)
1-3 y college	23 (62.2)
Graduated high school	6 (16.2)
Not high school graduate	2 (5.4)
**Have children, n (%)**	30 (71.4)

**Table 2:** Mean Changes^a^ in Metabolic and Body Composition Measures at Follow-Up Among 42 Urban American Indian Women With Impaired Fasting Glucose at Baseline in a Southwestern U.S. City, 2002–2006

Characteristic	Baseline Value, Mean (SD) n = 42	6-Month Change From Baseline, Mean (SD) n = 33	12-Month Change From Baseline, Mean (SD) n = 33	18-Month Change From Baseline, Mean (SD)n = 30	Overall Change Over Time*P* Value^b^
Fasting blood glucose, mmol/L	5.87 (0.32)	-0.22 (0.30)^c^	-0.19 (0.50)^c^	-0.39 (0.60)^c^	<.001
Diastolic blood pressure, mm Hg	71.8 (9.4)	-1.9 (8.3)	-1.0 (10.3)	-1.6 (8.2)	.54
Systolic blood pressure, mm Hg	119.3 (11.6)	+0.0 (11.0)	-3.8 (12.8)	-2.6 (12.5)	.21
Body mass index, kg/m^2^	34.1 (7.0)	-0.2 (1.7)	-0.5 (2.7)	-0.2 (2.8)	.60
Weight, kg	88.6 (17.5)	-0.5 (4.5)	-1.1 (6.9)	-0.5 (7.2)	.67
Waist circumference, cm	103.3 (15.5)	-1.4 (6.4)	-1.9 (6.3)	-2.2 (5.8)^c^	.23
Body fat, %	43.85 (6.00)	+0.12 (1.70)	+0.22 (2.03)	+0.84 (2.26)	.18
Triglycerides, mmol/L	1.81 (0.84)	-0.12 (0.54)	-0.18 (0.63)	-0.14 (0.47)	.40
Total fasting cholesterol, mmol/L	4.42 (0.81)	-0.21(0.58)^c^	-0.24 (0.58)^c^	-0.10 (0.50)	.03
LDL, mmol/L	2.48 (0.62)	-0.17 (0.48)	-0.23 (0.45)^c^	-0.09 (0.46)	.009
HDL, mmol/L	1.11 (0.20)	+0.02 (0.14)	+0.07 (0.15)	+0.05 (0.19)	.07

LDL indicates low-density lipoprotein cholesterol; HDL, high-density lipoprotein cholesterol.

a Follow-up value minus baseline value for each woman summed and averaged.

b Repeated measures analyses of variance to test for change over all 4 clinic visits.

c Significant difference from baseline value per paired *t* test, *P* < .05.

**Table 3:** Mean Changes^a^ in Dietary Intake, Activity, and Fitness at Follow-Up Among 42 Urban American Indian Women With Impaired Fasting Glucose at Baseline in a Southwestern U.S. City, 2002–2006

Characteristic	Baseline Value, Mean (SD) n = 42	6-Month Change From Baseline, Mean (SD) n = 33	12-Month Change From Baseline, Mean (SD) n = 33	18-Month Change From Baseline, Mean (SD) n = 30	Overall Change Over Time *P* value^b^
Total energy intake, kcal/day	2307.1 (971.6)	-349.2 (958.4)	-550.5 (683.2)^c^	-565.5 (853.6)^c^	< .001
Total fat intake, g/day	100.0 (45.6)	-20.5 (46.6)^c^	-20.4 (22.6)^c^	-24.2 (48.3)^c^	.004
Saturated fat intake, g/day	31.1 (15.1)	-6.2 (16.1)	-6.1 (6.8)^c^	-7.7 (16.0)^c^	.006
Proportion of sweet foods in diet, %	14.2 (7.0)	-1.7 (7.6)	-3.7 (10.5)	-5.2 (7.4)^c^	.01
Total sugar intake, g/day	111.2 (66.8)	-37.3 (78.3)^c^	-29.2 (77.9)^c^	-24.2 (81.7)	.03
Intake of sweetened beverages, oz/day	24.3 (20.0)	-11.32 (20.8)^c^	-7.17 (20.1)^c^	-5.12 (30.3)	.02
Peak VO_2,_ L/min	1.99 (0.43)	-0.13 (0.51)	-0.10 (0.48)	-0.15 (0.57)	.26
Total leisure-time physical activity, h/wk	4.4 (3.8)	+0.1 (4.1)	+0.3 (4.6)	+0.7 (4.7)	.76
Television watching, h/day	2.3 (2.1)	-0.6 (2.1)	-0.9 (1.9)^c^	-0.6 (2.0)	.03

VO_2_ indicates oxygen consumption.

a Follow-up value minus baseline value for each woman summed and averaged.

b Repeated measures analyses of variance to test for change over all 4 clinic visits.

c Significant difference from baseline value per paired *t* test, *P* < .05.
